# Allogeneic Splenocyte Transfer and Lipopolysaccharide Inhalations Induce Differential T Cell Expansion and Lung Injury: A Novel Model of Pulmonary Graft-versus-Host Disease

**DOI:** 10.1371/journal.pone.0097951

**Published:** 2014-05-20

**Authors:** Tereza Martinu, Christine V. Kinnier, Jesse Sun, Francine L. Kelly, Margaret E. Nelson, Stavros Garantziotis, W. Michael Foster, Scott M. Palmer

**Affiliations:** 1 Department of Medicine, Duke University Medical Center, Durham, North Carolina, United States of America; 2 Department of General Surgery, Massachusetts General Hospital, Boston, Massachusetts, United States of America; 3 School of medicine, University of North Carolina, Chapel Hill, North Carolina, United States of America; 4 Respiratory Biology Branch, National Institutes of Environmental Health Sciences, Research Triangle Park, North Carolina, United States of America; Cedars-Sinai Medical Center, United States of America

## Abstract

**Background:**

Pulmonary GVHD (pGVHD) is an important complication of hematopoietic cell transplant (HCT) and is thought to be a consequence of the HCT conditioning regimen, allogeneic donor cells, and posttransplant lung exposures. We have previously demonstrated that serial inhaled lipopolysaccharide (LPS) exposures potentiate the development of pGVHD after murine allogeneic HCT. In the current study we hypothesized that allogeneic lymphocytes and environmental exposures alone, in the absence of a pre-conditioning regimen, would cause features of pGVHD and would lead to a different T cell expansion pattern compared to syngeneic cells.

**Methods:**

Recipient Rag1^−/−^ mice received a transfer of allogeneic (Allo) or syngeneic (Syn) spleen cells. After 1 week of immune reconstitution, mice received 5 daily inhaled LPS exposures and were sacrificed 72 hours after the last LPS exposure. Lung physiology, histology, and protein levels in bronchoalveolar lavage (BAL) were assessed. Lung cells were analyzed by flow cytometry.

**Results:**

Both Allo and Syn mice that undergo LPS exposures (AlloLPS and SynLPS) have prominent lymphocytic inflammation in their lungs, resembling pGVHD pathology, not seen in LPS-unexposed or non-transplanted controls. Compared to SynLPS, however, AlloLPS have significantly increased levels of BAL protein and enhancement of airway hyperreactivity, consistent with more severe lung injury. This injury in AlloLPS mice is associated with an increase in CD8 T cells and effector CD4 T cells, as well as a decrease in regulatory to effector CD4 T cell ratio. Additionally, cytokine analysis is consistent with a preferential Th1 differentiation and upregulation of pulmonary CCL5 and granzyme B.

**Conclusions:**

Allogeneic lymphocyte transfer into lymphocyte-deficient mice, followed by LPS exposures, causes features of pGVHD and lung injury in the absence of a pre-conditioning HCT regimen. This lung disease associated with an expansion of allogeneic effector T cells provides a novel model to dissect mechanisms of pGVHD independent of conditioning.

## Introduction

Pulmonary complications after hematopoietic-cell transplant (HCT) are an important cause of morbidity and mortality. Non-infectious pulmonary complications are thought to be a manifestation of pulmonary graft-versus-host disease (pGVHD) but are poorly understood and difficult to treat [1]–[3]. In fact, it is unclear why some patients recover well from HCT but later develop pGVHD. It is postulated that the constant exposure to the environment potentiates innate immune pathways in the lungs and augments pGVHD. Lymphocytic bronchiolitis (LB), airway obstruction, and long-term development of fibrotic airway obliteration are features of pGVHD [4], [5].

Our laboratory has focused on the role of environmental stimuli as triggers of pGVHD. We have previously demonstrated that, in mice recipient of allogeneic HCT, inhaled LPS, as a prototypic innate immune stimulus, potentiates pGVHD [6], [7]. The low grade LPS exposures used in our HCT model replicate human airway gram-negative bacterial colonization as well as workplace and domestic environmental exposures [8], [9]. It is assumed that the pre-conditioning HCT regimen, including chemotherapy and radiation, and not only the presence of allogeneic cells, contribute to systemic GHVD as well as pGVHD. However, given that pGVHD often develops much later than and independently of systemic GHVD, we postulated that pGVHD can develop without a conditioning regimen. We hypothesized that allogeneic lymphocytes by themselves, without irradiation or chemotherapy, are capable of causing features of pGVHD in the setting of an environmental trigger.

In this study, we demonstrate that transfer of allogeneic splenocytes into lymphopenic Rag1^−/−^ mice, followed by serial pulmonary LPS exposures, leads to more severe airway injury and lymphocytic bronchiolitis, consistent with pGVHD. This lung injury pattern is associated with increased CD8 T cells and increased effector CD4 T cells.

## Materials and Methods

### Ethics Statement

All experiments were approved by the Institutional Animal Care and Use Committees at Duke University (protocol number A056-09-02) and strictly followed the National Institutes of Health recommendations cited in the Guide for the Care and Use of Laboratory Animals. All potentially painful procedures were performed under isoflurane anesthesia and all efforts were made to minimize suffering.

### Mice

Male 6–8 week old B6.129S7-Rag1^tm1Mom^/J (Rag1^−/−^, H2D^b^), CD45.1-expressing B6. SJL-Ptprc^a^Pepc^b^/BoyJ (B6, H2^b^), and C3HeB/FeJ (B/Fe, H2^k^) mice were purchased from Jackson Laboratories (Bar Harbor, ME). All animals were housed in a pathogen-free facility at Duke University on LPS-free bedding (Alpha Dri bedding, Shepherd Specialty Papers Inc., Kalamazoo, MI) and were fed irradiated food (PicoLab Mouse Diet 20 5058, Purina Mills, Richmond, IN).

### Splenocyte Transfer

Donor B6 and B/Fe mice were euthanized using CO_2_. Splenocytes were isolated from their spleens *via* homogenization and filtration. All donor cells were washed in media, filtered through 70 um filters (BD, Franklin Lakes, NJ), counted on a hemocytometer, and resuspended at an appropriate concentration in media containing 10% FBS (Hyclone, Logan, UT), 1% L-Glutamine (Sigma-Aldrich, St. Louis, MO) and 1% Penicillin/Streptomycin (Sigma-Aldrich). Rag1^−/−^ recipient mice were injected intravenously *via* the retro-orbital route with 5×10^6^ donor splenocytes in a total volume of 0.5 mL.

### LPS Exposures

LPS exposures, starting 1 week after splenocyte transfer, were performed by aerosol inhalation using lyophilized LPS from *E. coli* 0111:B4 (Sigma-Aldrich, St. Louis, MO). The LPS solution was prepared as previously described [10]. Aerosol was delivered to a 20 L inhalation chamber using a constant-output six-jet atomizer model 9306 (TSI Inc., Shoreview, MN) at 35 psi, which generates aerosol droplets with a mean diameter of 0.5 um at a flow rate of about 3.3 L/min. This achieves a final LPS chamber concentration of approximately 4.5 ug/m^3^ [10], [11]. Mice were placed into stainless steel caging and put into the inhalation chamber and exposed to LPS for 2.5 hours/day for 5 consecutive days. Mice were euthanized either without LPS exposure or 72 hours after the final LPS exposure.

### Bronchoalveolar Lavage (BAL)

Mice were euthanized using CO_2_ and their lungs were surgically exposed. The trachea was cannulated and the lungs were lavaged with 5 aliquots of 800 µL of 0.9% saline (VWR International, West Chester, PA). Bronchoalveolar lavage (BAL) fluid supernatant was stored at −80°C until further use. BAL fluid total protein was quantified with a total protein assay kit using Bicinchoninic Acid (Thermo Scientific, Waltham, MA) and Granzyme B was measured using a specific ELISA kit (R&D systems, Minneapolis, MN). Cytokine analysis was performed on the BAL fluid using mouse 23-plex and 9-plex cytokine assays that included detection of IFN-γ, IL-4, IL-5, IL-13, IL-17, IL-10, IL-15, and CCL5 (Bio-Rad Laboratories, Hercules, CA).

### Lung Tissue Extraction and Processing

After BAL, lungs were perfused with 0.9% saline. The right lung was placed in a HEPES-based buffer (Sigma-Aldrich) for subsequent processing for flow cytometry. The left lung was gravity-inflated with 10% formalin (VWR International), fixed in 10% formalin solution for 24 hours, and then transferred into 70% EtOH. The left lung was subsequently embedded in paraffin and 5 µm sections were stained with H&E for histologic analysis. Pathological severity of perivascular and peribronchiolar lymphocytic lung inflammation was graded on a 9-point scale as described previously [6]. Additional sections were stained with rabbit anti-CD3 (Lab Vision Corp, Fremont, CA) (diluted 1∶100). Rabbit Ig (diluted 1∶60,000, Dako USA, Carpinteria, CA) was used as negative control. Primary antibody was detected with anti-rabbit horseradish-peroxidase (Dako USA).

### Lung Flow Cytometry

Fresh lung tissue was homogenized, digested with 1 mg/mL collagenase A (Roche Diagnostics, Mannheim, Germany) and 0.2 mg/mL DNAse I (Sigma-Aldrich), filtered through a 70 um filter (BD), red cell lysed and washed. Lung cells were resuspended in 500 µL of flow cytometry buffer containing phosphate-buffered sodium solution (Sigma-Aldrich) with 3% FBS (Hyclone), 0.05% sodium azide (VWR International), and 10 mM EDTA (Sigma-Aldrich). Cells were blocked for 10 minutes using flow cytometry buffer with the addition of 5% normal mouse serum, 5% normal rat serum, and 1% F_c_ receptor block (Affinity purified anti-mouse CD16/32) (BioLegend, San Diego, CA). Cells were subsequently stained with conjugated antibodies for 30 minutes. The flow cytometry lymphocyte staining panel included FITC-conjugated anti-mouse CD3, PE-Cy7-conjugated anti-mouse CD8, APC-Cy7-conjugated anti-mouse CD4 (BioLegend), PE-conjugated anti-mouse CD44, APC-conjugated anti-mouse CD62L (BD Biosciences, San Jose, CA), and PerCP-Cy5.5-conjugated anti-mouse CD25 (eBioscience, San Diego, CA). The myeloid staining panel included FITC-conjugated anti-mouse MHCII, PE-conjugated anti-mouse H2K^k^ or CD45.1 (BD Bioscience), PE-Cy7-conjugated anti-mouse CD11b, PerCP-Cy5.5-conjugated anti-mouse CD45 (BioLegend), and APC-conjugated anti-mouse CD11c (eBioscience). The natural killer (NK) cell staining panel included the NK-specific APC-conjugated anti-mouse CD49b (clone DX5) (eBioscience). The FOXP3 Transcription Factor Staining Buffer set (eBioscience) was used for intracellular staining with PE-conjugated anti-mouse FOXP3 along with extracellular staining using APC-conjugated anti-mouse CD25, PerCP-Cy5.5-conjugated anti-mouse CD4 (eBioscience), PE-Cy7-conjugated anti-mouse CD3, and APC-Cy7-conjugated anti-mouse CD8 (BioLegend). Cell fluorescence was measured using a BD FACS Canto II flow cytometer with BD FACSDiva software (BD). Flow cytometry analysis was performed using FlowJo software (Tree Star Inc., Ashland, OR). A singlet gate (based on forward scatter height by area plot) was used to exclude cell aggregates, followed by an all-cell gate (based on forward scatter by side scatter plot) to exclude small debris and dead cells, followed by a CD45^+^ cell gate to define all white blood cells. Percentage of all cells was converted to an absolute cell number by multiplying by the corresponding live-cell count.

### Airway Resistance Measurement

Lung physiology measurements were performed *in vivo* using the *flexi*Vent mechanical ventilator and data acquisition system (SCIREQ, Montreal, PQ, Canada). Mice were anesthetized (Nembutal, 60 mg/kg, IP), then paralyzed with pancuronium bromide (0.82 mg/kg, IP), and a tracheal cannula was inserted. A differential pressure transducer was connected to the tracheal cannula and ventilation was maintained at a rate of 120 breaths/minute, with a tidal volume of ∼8 ml/kg and a positive end-expiratory pressure of ∼3 cmH2O. Values for total resistance (R) and for total lung compliance were obtained over 6 cycles of total lung capacity maneuvers. To assess airway responsivity, mice were challenged with increasing doses of aerosolized methacholine (0.0–100.0 mg/ml) as previously described [12]. Values were averaged for each animal.

### Statistical Analysis

Data are expressed as means ± SEM. Individual comparisons between groups were performed using a Student’s t-test. Curves for airway resistance in response to increasing doses of methacholine were compared using a two-way ANOVA for repeated measures analysis. Throughout the graphs, star (*) indicates a p-value of <0.05, two stars (**) p<0.005, and three stars (***) p<0.0005. Experimental groups included 3 to 10 animals and experiments were performed at least twice.

## Results

### Splenocyte Transfer into Lymphopenic Rag1^−/−^ Mice Followed by Inhaled LPS Leads to pGVHD Pathology

**Figure 1:**
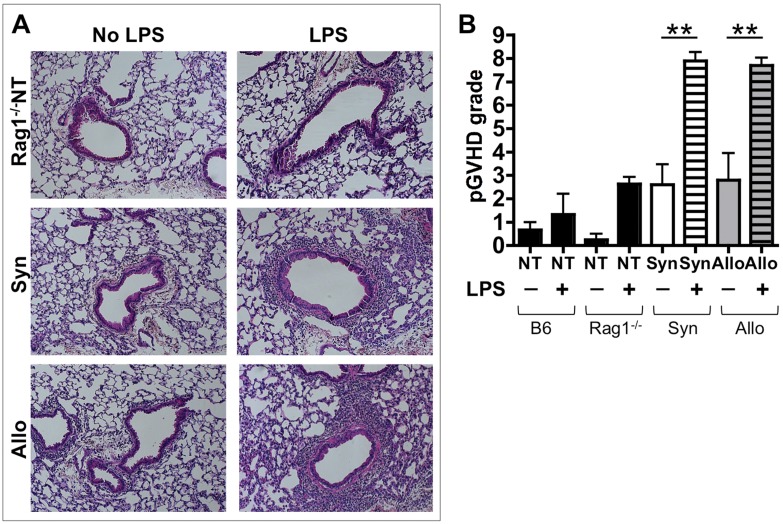
Splenocyte transfer followed by inhaled LPS leads to pGVHD pathology. Rag1^−/−^ mice received allogeneic (Allo) or syngeneic (Syn) splenocytes or no splenocyte transfer (Rag1^−/−^NT). Additional wild-type C57BL/6 (B6) mice without splenocyte transfer (B6NT) were used as controls. Allo, Syn, Rag1^−/−^NT and B6NT mice underwent daily exposures to aerosolized LPS for 5 days starting 1 week after splenocyte transfer. Mice were euthanized 72 hours after the last LPS exposure. (**A**) Lung pathology assessment shows perivascular and peribronchiolar mononuclear inflammation in the AlloLPS and SynLPS mice *(H&E, 100X)*. Only minimal inflammation is seen in AlloNoLPS and SynNoLPS lungs. Rag1^−/−^NT mouse lung pathology is shown for additional comparison and is similar to that of B6NT mouse lungs. After LPS, all NT mice have rare mononuclear cells visible in the perivascular and peribronchiolar structures. This is similar to pathology seen in B6NT mice. (**B**) Lung pGVHD pathology was graded in a blinded fashion using a 0–9 semi-quantitative grading schema to express the thickness of the mononuclear infiltrate around airways and around vessels as well as the overall extent of the pathology in the lung. SynLPS and AlloLPS lungs have a grade of about 8, which is significantly higher than the grade of non-LPS exposed controls where the grade is about 2.5 (AlloLPS vs. AlloNoLPS p = 0.003 and SynLPS vs. SynNoLPS p = 0.0005). As measured by this grading, LPS led to low-grade background inflammation in NT mice as shown in the graph. Data represent the average +/− SEM and **represents p<0.005. Data have been replicated in 3 independent experiments.

We had previously demonstrated that murine allogeneic HCT, which uses irradiation pre-conditioning followed by inhaled LPS (iLPS) exposures, leads to pGVHD similar to that seen in humans [6], [7]. We set off to assess the effect of allogeneic lymphocytes on LPS-exposed lungs in the absence of irradiation injury. Allogeneic (Allo) and Syngeneic (Syn) splenocytes were transferred into Rag1^−/−^ lymphopenic mice. Two weeks post splenocyte transfer, Allo and Syn mice have 100% survival and no signs of systemic GVHD such as weight loss, diarrhea, or skin changes (data not shown). At two weeks post splenocyte transfer, without any LPS exposures, the Allo and Syn mice also have minimal pulmonary pathology with rare lymphocytes in the perivascular and peribronchiolar spaces (figure 1A&B). One week after splenocyte transfer, Allo and Syn mice underwent 5 daily iLPS exposures (AlloLPS mice and SynLPS) and their lungs were assessed 72 hours after the last iLPS exposure. Assessment of pulmonary pathology demonstrates that splenocyte transfer followed by iLPS leads to lymphocytic inflammation, preferentially located around the airways, similar to pGVHD in our murine HCT model as well as in humans (figure 1A). Surprisingly, this lymphocytic inflammation is similar in AlloLPS and SynLPS mice (figure 1A&B). To demonstrate that this is not a normal pulmonary response to sub-acute iLPS, non-transplanted (NT) Rag1^−/−^ mice (figure 1A), as well as non-transplanted (NT) wild-type C57BL/6 (B6) mice underwent the same iLPS exposures. Strikingly, both sets of non-transplanted mice have minimal inflammation after sub-acute iLPS and do not show pGVHD pathology (figure 1B).

### Only Allogeneic Splenocyte Transfer Followed by Inhaled LPS Leads to Increased Lung Injury

**Figure 2:**
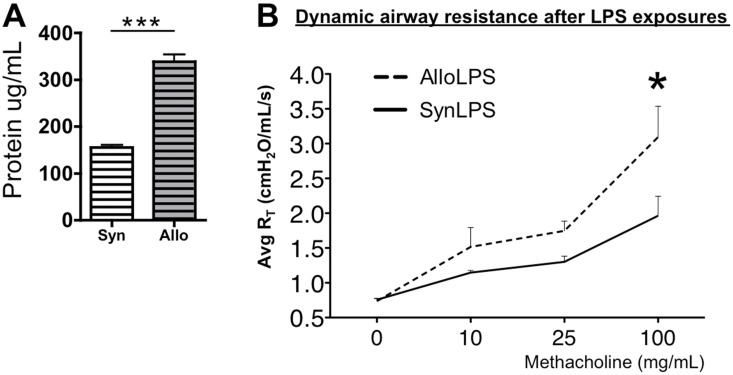
Allogeneic splenocyte transfer followed by inhaled LPS leads to lung injury and airway hyperreactivity (AHR). Rag1^−/−^ mice received allogeneic (Allo) splenocytes or syngeneic (Syn) splenocytes and underwent daily exposures to aerosolized LPS for 5 days starting 1 week after splenocyte transfer. Airway physiology was studied and mice were euthanized 72 hours after the last LPS exposure. (**A**) BAL fluid protein levels were measured and were found to be significantly higher in AlloLPS mice as compared to SynLPS (p<0.0001). (**B**) Airway resistance was measured *in vivo* in response to increasing doses of aerosolized methacholine. Airway resistance in AlloLPS mice was significantly enhanced in dose-related manner as compared to AlloNoLPS and SynLPS controls (p = 0.045). Data represent the average +/− SEM and * = p<0.05, *** = p<0.0005. Data have been replicated in 2 independent experiments.

We assessed whether in the setting of iLPS, in spite of similar pGVHD pathology, allogeneic splenocyte transfer into Rag1^−/−^ mice (AlloLPS) leads to more lung disease compared to the syngeneic controls (SynLPS). BAL total protein levels are increased in AlloLPS mice compared to SynLPS (figure 2A), demonstrating a loss of epithelial integrity and increased lung injury. Additionally, there is increased airway hyperresponsiveness (AHR) to aerosolized methacholine in AlloLPS mice as compared to the SynLPS controls (figure 2B), also consistent with injury and alteration of airway function. The overall lung tissue compliance is not different between AlloLPS and SynLPS (data not shown), suggesting minimal injury to the alveolar and interstitial lung compartments, consistent with a preferential injury to the airways.

### After Allogeneic Lymphocyte Transfer Followed by Inhaled LPS, Pulmonary Donor-derived Cells are Comprised Primarily of Lymphocytes While Myeloid Cells are of Recipient Origin

**Figure 3:**
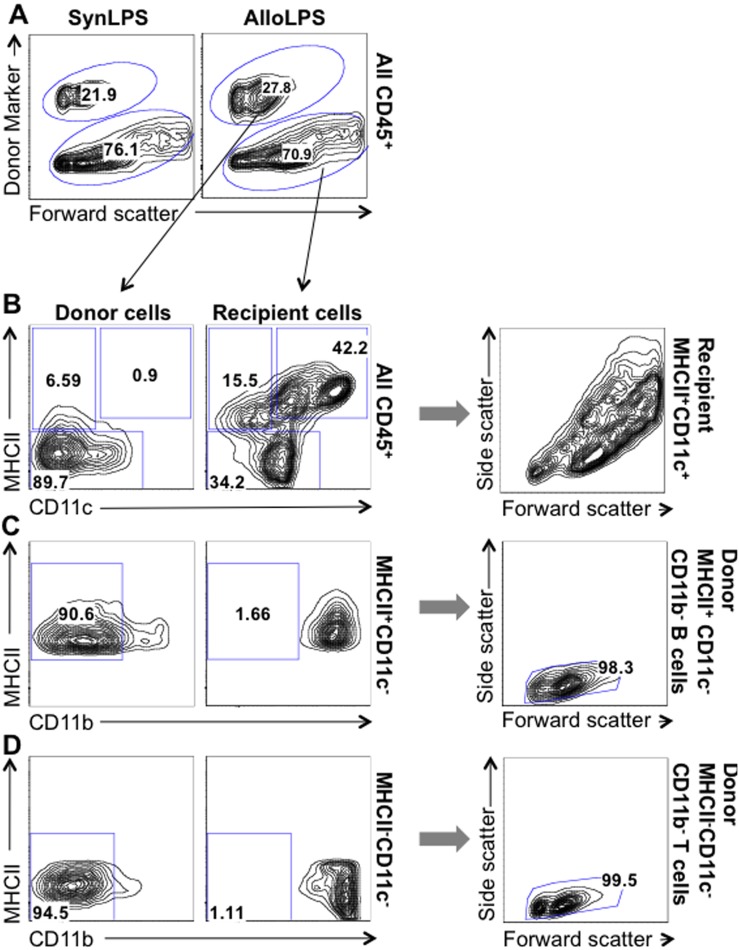
After allogeneic lymphocyte transfer followed by inhaled LPS, pulmonary donor-derived cells are comprised primarily of lymphocytes while myeloid cells are of recipient origin. Rag1^−/−^ mice received a transfer of allogeneic (Allo) or syngeneic (Syn) splenocytes and underwent daily exposures to aerosolized LPS for 5 days starting 1 week after splenocyte transfer. Mice were euthanized 72 hours after the last LPS exposure and lung cells were analyzed using flow cytometry. For flow analysis, a singlet gate was used to exclude cell aggregates, followed by an all-cell gate to exclude small debris and dead cells, followed by a CD45^+^ cell gate to define all white blood cells (as described in methods). (**A**) All CD45^+^ cells were separated into donor and recipient-derived cells based on their expression, or lack thereof, of the donor marker CD45.1 (in the case of SynLPS mice on the left) or H2Kk (in the case of AlloLPS mice on the right). The subsequent graphs show flow cytometry plots for AlloLPS lung cells but the same analysis was performed for SynLPS and yielded similar results. (**B**) Donor- (left) and recipient-derived (right) lung cells were analyzed separately. Representative flow cytometry plots show gating of MHC^+^CD11c^+^ antigen-presenting myeloid cells, which are mainly present in the recipient cells (42.2% vs. 0.9% in donor cells) and are large based on the side by forward scatter graph (far right). The MHCII^+^CD11c^−^ cells are classically comprised of B cells and CD11b^+^MHCII^+^ myeloid cells. MHCII^−^CD11c^−^ cells are usually comprised of neutrophils, monocytes, and T cells. (**C**) Representative flow cytometry plots show the population of MHCII^+^CD11c^−^ cells and gating of the CD11b^−^ B cells that are more abundant among donor cells compared to recipient cells (90.6% vs. 1.66%). The far right graph, showing a side by forward scatter plot, demonstrates that these donor MHCII^+^CD11c^−^CD11b^−^ B cells are indeed small cells. (**D**) Representative flow cytometry plots show the population of MHCII^−^CD11c^−^ cells and gating of the CD11b^−^ T cells that are more abundant among donor cells compared to recipient cells (94.5% vs. 1.11%). The far right graph, showing a side by forward scatter plot, again demonstrates that these donor MHCII^−^CD11c^−^CD11b^−^ T cells are indeed small cells.

Splenocytes are predominantly comprised of lymphocytes. While there is a small percentage of myeloid cells in the spleen single-cell suspension used for reconstitution of Allo and Syn mice, we find almost no donor-derived myeloid cells in the lungs of AlloLPS and SynLPS mice (figure 3A&B). Myeloid cells are defined as CD45^+^MHCII^+^CD11c^+^ cells and CD45^+^CD11b^+^ cells (figure 3B–D) as previously described in the literature [13], [14]. The donor-derived lung cells at the 2-week time point in AlloLPS and SynLPS mice are essentially all lymphocytes, defined as CD45^+^ MHCII^+^CD11c^−^CD11b^−^small B cells (figure 3B&C) and CD45^+^MHCII^−^CD11c^−^CD11b^−^small T cells (figure 3B&D). The characterization of B cells as CD45^+^MHCII^+^CD11c^−^CD11b^−^small cells was used because prior studies reported that Rag1^−/−^ mice have only immature B cells with low expression of classic B cell markers such as B220 [15]. Figure 3 shows representative flow cytometry plots of lung cells from AlloLPS mice where donor cells are based on their expression of H2K^k^. The same analysis was performed for SynLPS, using the donor marker CD45.1, and yielded similar results.

### Allogeneic Lymphocyte Transfer Followed by Inhaled LPS Results in Increased Pulmonary CD8 T Cells

**Figure 4:**
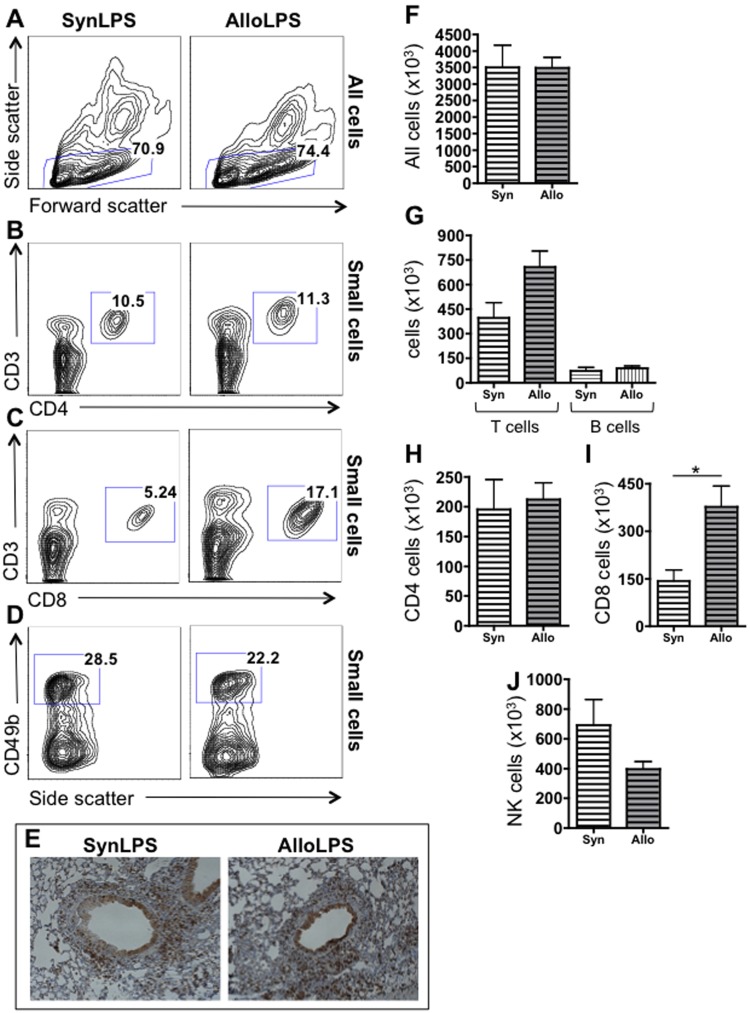
Allogeneic splenocyte transfer followed by inhaled LPS leads to preferential expansion of CD8 T cells. Rag1^−/−^ mice received a transfer of allogeneic (Allo) or syngeneic (Syn) splenocytes and underwent daily exposures to aerosolized LPS for 5 days starting 1 week after splenocyte transfer. Mice were euthanized 72 hours after the last LPS exposure and lung lymphocytes were analyzed using flow cytometry and immunohistochemistry. Representative flow cytometry plots for SynLPS and AlloLPS show the gating of (**A**) small cells among all cells, (**B**) CD3 versus CD4 gating among small cells, (**C**) CD3 versus CD8 gating among small cells where the percentage of CD8 T cells is higher in AlloLPS (17.1%) compared to SynLPS (5.24%), and (**D**) gating of CD49^+^ NK cells among small cells. (**E**) Lung immunohistochemical staining for the CD3 antigen demonstrates that CD3 T cells represent the majority of cells in the infiltrates around airways and blood vessels in AlloLPS and SynLPS mice. (**F**) By flow cytometry, there is no difference in numbers of total cells between the lungs of AlloLPS and SynLPS mice. (**G**) Total CD3 T cells or B cells are also not different between the lungs of AlloLPS and SynLPS mice. It is apparent, however, that CD3 T cells are much more abundant than B cells in both AlloLPS and SynLPS. (**H–I**) Further analysis of T cells by flow cytometry shows that CD8 T cells are significantly increased in the lungs of AlloLPS mice as compared to SynLPS (p = 0.03), while CD4 T cells are unchanged. (**J**) NK cell numbers, as identified by CD49b expression, are not significantly different between AlloLPS and SynLPS mice. Numeric data represent the average +/− SEM and * = p<0.05. Data have been replicated in 2 independent experiments.

The total number of cells is not different between AlloLPS and SynLPS lungs (figure 4F), consistent with the similarities seen on gross pathology. To elucidate the mechanism that may be responsible for the more severe lung injury (characterized by increased AHR and BAL protein) after allogeneic splenocyte transfer and LPS, we focused on characterizing the exact composition of the cellular infiltrates specifically between AlloLPS and SynLPS mice. Because the donor-derived cells in the lungs of AlloLPS and SynLPS mice are primarily lymphocytes (figure 3), we undertook a detailed analysis of lymphocyte sub-populations. The lymphocytic inflammation in both AlloLPS and SynLPS lungs is comprised mostly of CD3^+^ T cells (figure 4G, gating based on either 4B, 4C, or 3B&D give the same results), rather than B cells (figure 4G, gating shown in figure 3B&C), as seen by flow cytometric analysis. Furthermore, CD3^+^ T cells are abundant in the peribronchiolar space of AlloLPS and SynLPS mice by immunohistochemistry (figure 4E). When specifically analyzing T cell sub-populations, there is no significant difference in total CD4 T cells between AlloLPS and SynLPS (figure 4B&H). However, CD8 T cells are significantly elevated in the lungs of AlloLPS mice compared to SynLPS (figure 4C&I). Of note, CD49^+^ NK cells are not significantly different between AlloLPS and SynLPS (figure 4D&J).

### Allogeneic Lymphocyte Transfer Followed by Inhaled LPS Leads to Expansion of CD8 T Cell Subsets and Preferential Expansion of CD4 Effector T Cells

**Figure 5:**
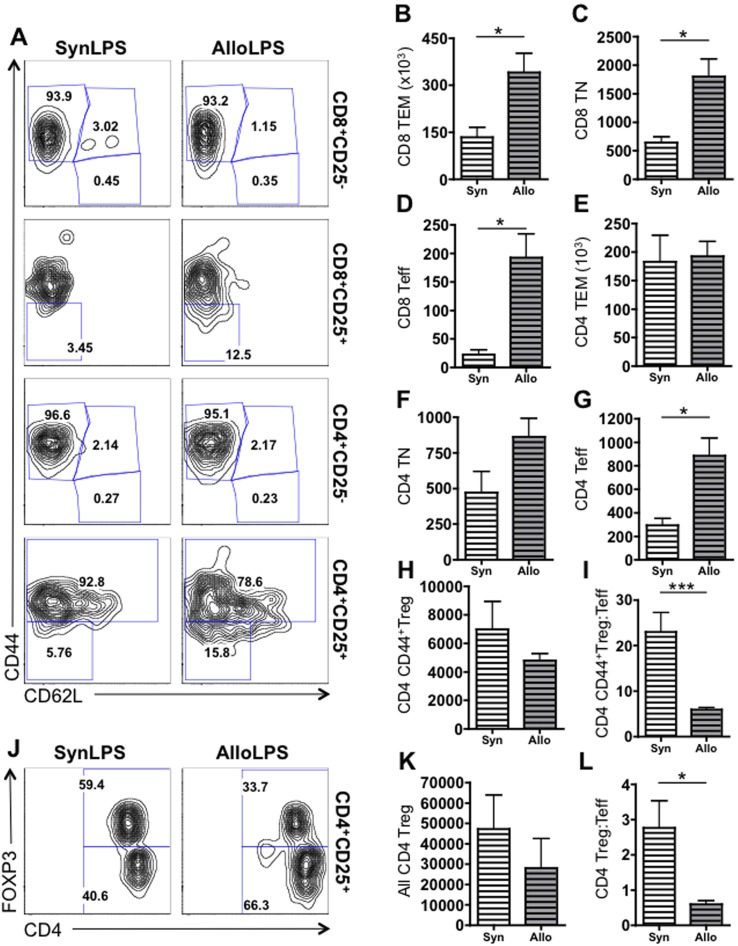
Allogeneic splenocyte transfer followed by inhaled LPS leads to preferential expansion of all CD8 T cell subsets and of effector CD4 T cells. Rag1−/− mice received a transfer of allogeneic (Allo) or syngeneic (Syn) splenocytes followed by daily exposures to aerosolized LPS for 5 days starting 1 week after splenocyte transfer. Mice were euthanized 72 hours after the last LPS exposure and lung cells were analyzed using flow cytometry. CD8 and CD4 T cells were gated as described in figure 4 and then further subdivided into CD25+ and CD25− cells. (A) Representative flow cytometry plots for lungs cells from SynLPS (left column) and AlloLPS (right column) show expression of CD44 and CD62L. In the first row, CD8+CD25− cells are subdivided in CD44+CD62L− effector memory CD8 T cells (CD8 TEM), CD44+CD62L+ central memory CD8 T cells (CD8 TCM), and CD44−CD62L+ naïve CD8 T cells (CD8 TN). The majority of these CD25− CD8 T cells are TEM cells. In the second row, the subset of activated CD8 effector T cells (CD8 Teff), which are negative for CD44 and CD62L, is shown as a percentage of all activated CD8+CD25+ cells. The CD8 Teff percentage is higher in AlloLPS lungs as compared to the SynLPS lungs. In the third row, CD4+CD25− cells are subdivided in CD44+CD62L− effector memory CD4 T cells (CD4 TEM), CD44+CD62L+ central memory CD4 T cells (CD4 TCM), and CD44−CD62L+ naïve CD4 T cells (CD4 TN). The majority of these CD25− CD4 T cells are TEM cells. In the fourth row, the subset of activated CD4 effector T cells (CD4 Teff), which are negative for CD44 and CD62L, is shown as a percentage of all activated CD4+CD25+ cells. The CD4 Teff percentage is higher in AlloLPS lungs as compared to the SynLPS lungs. The CD44+ sub-population of CD4+CD25+ activated T cells have been previously identified as regulatory T cells (Treg). The percentage of these CD4 CD44+Treg is lower in AlloLPS compared to SynLPS. (B–I&K–L) Quantitative graphs are shown of absolute numbers of T cells in lungs of AlloLPS and SynLPS mice. (B–D) CD8 TEM (p = 0.038), CD8 TN (p = 0.025), and CD8 Teff (p = 0.015) cell numbers are all increased in AlloLPS lungs compared to SynLPS. (E–F) CD4 TEM and CD4 TN numbers are similar between AlloLPS and SynLPS lungs. (G) CD4 Teff cells are significantly increased in AlloLPS compared to SynLPS lungs (p = 0.019). (H) While CD4 CD44+Treg cell numbers are similar between AlloLPS and SynLPS, (I) the ratio of CD44+Treg to Teff is significantly lower in AlloLPS (p<0.0001). (J) Representative flow cytometry plots for CD4+CD25+ lungs cells from SynLPS (left) and AlloLPS (right) show expression of intracellular FOXP3 and CD4. The percentage of FOXP3+CD4+CD25 regulatory T cells (Treg) is lower, while the percentage of FOXP3−CD4+CD25+ effector T cells (Teff) is elevated, in AlloLPS compared to SynLPS. (K) While FOXP3+CD4+CD25+ Treg numbers are similar between AlloLPS and SynLPS, (L) the ratio of FOXP3+CD4+CD25+ Treg to FOXP3−CD4+CD25+ Teff is significantly lower in AlloLPS (p = 0.049). Numeric data represent the average +/− SEM and * = p<0.05 and *** = p<0.0005.

To better understand the T cell expansion that occurs after inhaled LPS in Allo and Syn lungs, we assessed the expression of CD25, CD44, and CD62L to identify sub-populations of T cells [16]. We specifically identified CD25^−^CD44^+^CD62L^−^ effector memory T cells (TEM), CD25^−^CD44^−^CD62L^+^ naïve T cells (TN), CD25^+^CD44^−^CD62L^−^ effector T cells (Teff) within both CD4 and CD8 T lymphocytes. We also measured CD4^+^CD25^+^CD44^+^ regulatory T cells (CD44^+^Treg). We show that all sub-populations of CD8 T cells are elevated in AlloLPS lungs compared to SynLPS, including CD8 TEM (figure 5A&B), CD8 TN (figure 5A&C), and CD8 Teff (figure 5A&D). There is no significant change in CD4 TEM (figure 5A&E), CD4 TN (figure 5A&F), or CD4 CD44^+^Treg (figure 5A&H) between AlloLPS and SynLPS lungs. However, CD4 Teff are significantly elevated in AlloLPS lungs compared to SynLPS (figure 5A&G). Additionally, the ratio of CD44^+^Treg to Teff among CD4 T cells is significantly reduced in AlloLPS lungs (figure 5A&I). While CD4^+^CD25^+^CD44^+^ T cells are comprised almost exclusively of regulatory T cells based on recent reports [17], a more comprehensive way to identify regulatory CD4 T lymphocytes is by assessment of their expression of intracellular FOXP3. We therefore assessed the expression of CD4, CD25, and intracellular FOXP3 in the lung cells and found that there is no difference between CD4^+^CD25^+^FOXP3^+^ Treg between AlloLPS and SynLPS lungs (figure 5J&K). However, consistent with the prior staining strategy, the ratio of CD4^+^CD25^+^FOXP3^+^ Treg to the CD4^+^CD25^+^FOXP3^−^ Teff is reduced in AlloLPS lungs as compared to SynLPS (figure 5J&L). This suggests that in the context of alloantigen exposure and an environmental stimulus, there is preferential expansion of CD8 T cells and CD4 effector T cells out of proportion to CD4 regulatory T cells.

### Allogeneic Lymphocyte Transfer Followed by Inhaled LPS Leads to Increased IFN-γ, Granzyme B, and CCL5

**Figure 6:**
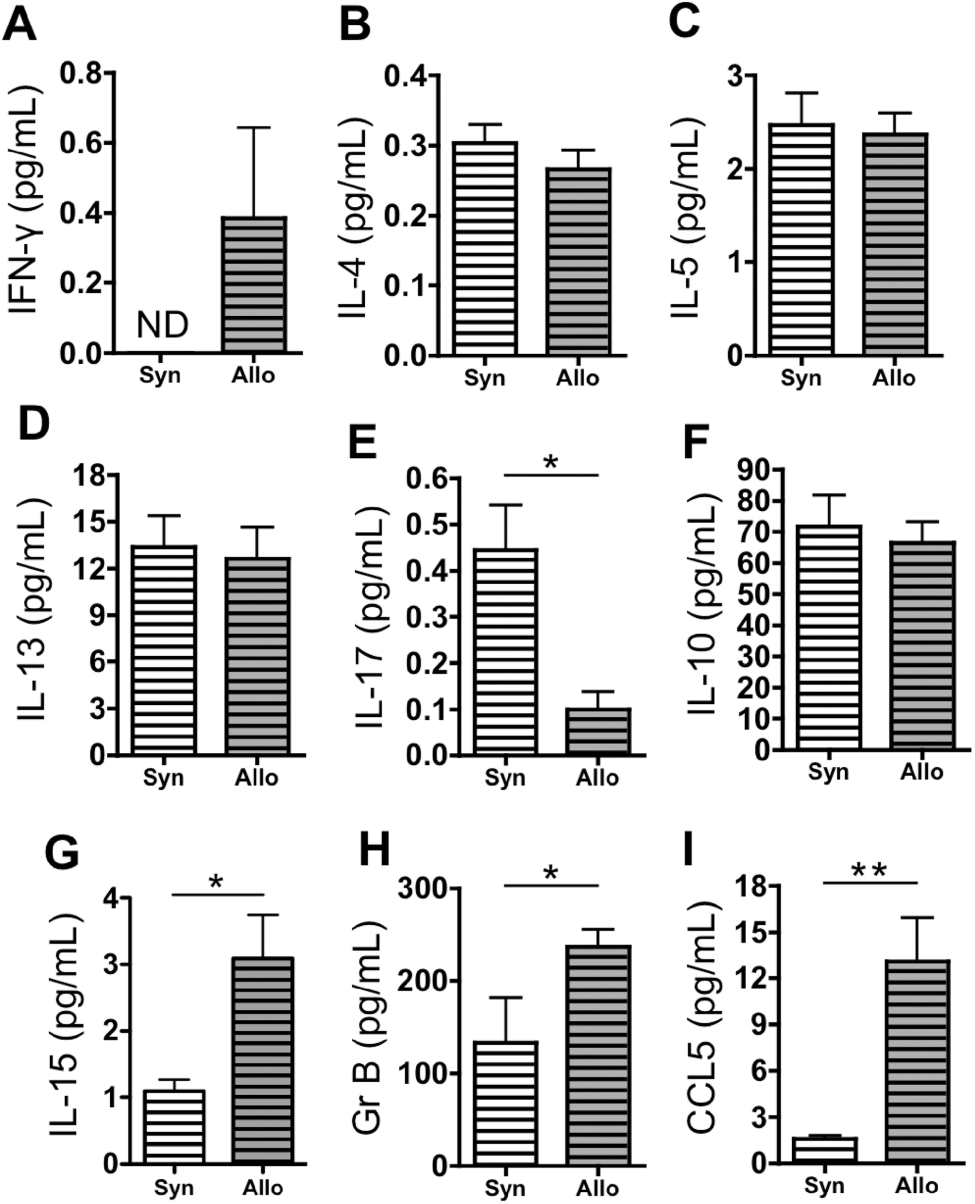
Allogeneic splenocyte transfer followed by inhaled LPS leads to an increase in pulmonary IFN-γ, granzyme B, and CCL5. Rag1^−/−^ mice received a transfer of allogeneic (Allo) or syngeneic (Syn) splenocytes followed by daily exposures to aerosolized LPS for 5 days starting 1 week after splenocyte transfer. Mice were euthanized 72 hours after the last LPS exposure and concentrations of proteins were measured in the BAL of AlloLPS and SynLPS mice using multiplex and ELISA assays. (**A**) IFN- γ is elevated in AlloLPS compared to SynLPS (p = ). (**B–D**) Th2 cytokines IL-4, IL-5, and IL-13 are similar between AlloLPS and SynLPS. (**E**) IL-17 is reduced in AlloLPS compared to SynLPS (p = 0.0078). (**F**) IL-10 is unchanged between AlloLPS and SynLPS. (**G**) IL-15 is elevated in the BAL of AlloLPS compared to SynLPS (p = 0.014). (**H**) Granzyme B (Gr B) is elevated in the BAL of AlloLPS mice compared to SynLPS (p = 0.029). (**I**) CCL5 is elevated in the BAL of AlloLPS mice compared to SynLPS (p = 0.0023). Data represent the average +/− SEM and * = p<0.05 and ** = p<0.005. ND = non detectable. Data have been replicated in 2 independent experiments.

We hypothesized that the T cell expansion in AlloLPS lungs, as compared to SynLPS lungs, induces lung injury and AHR through a specific cytokine pattern. We therefore measured a panel of cytokines in the BAL, including cytokines related to helper T cell differentiation as well as CD8-specific proteins. IFN-γ, the prototypic Th1 cytokine, is elevated in the BAL of AlloLPS mice compared to SynLPS (figure 6A), suggesting a preferential Th1 CD4 T cell differentiation. Th2 cytokines, IL-4, IL-5, and IL-13, are similar between AlloLPS and SynLPS (figure 6B–D). IL-17, the main Th17 cytokine, is reduced in AlloLPS compared to SynLPS (figure 6E) and IL-10, classically produced by Tregs, is unchanged (figure 6F). IL-15, known to promote CD8 T cell proliferation, is elevated in AlloLPS compared to SynLPS (figure 6G). Granzyme B and CCL5, proteins known to be produced preferentially by CD8 T cells, are increased in AlloLPS versus SynLPS lungs (figure 6H&I).

## Discussion

In this report, we demonstrate that lymphocyte transfer into lymphopenic mice, without any conditioning regimen, followed by sub-acute exposure to inhaled LPS, replicates the pathologic features of pGVHD seen in humans [4], [5] and in mouse models of this disease [6], [7]. Additionally, compared to syngeneic controls, allogeneic lymphocytes confer a more severe phenotype with increased AHR and alteration of epithelial integrity in this model. The AlloLPS lung phenotype is associated with preferential pulmonary expansion of CD8 T cells and an increase in CD4 effectors out of proportion to regulatory T cells.

In this novel system, using allogeneic splenocyte infusion into Rag1^−/−^ mice, our data suggest that that development of pGVHD requires the combination of at least two injurious events. Allogeneic lymphocytes by themselves do not cause any significant lung inflammation but rather require the addition of environmental exposures to result in disease. We show that this can occur independent of a conditioning regimen, such as irradiation or chemotherapy, used in prior models of pGVHD [6], [7], [18]. Furthermore, the prominent pathology of lymphocytic bronchiolitis in the absence of alveolar or interstitial inflammation, as well as the increase in dynamic airway resistance and normal lung compliance, point to preferential airway injury in this model. Our allogeneic pGVHD model is thus consistent with other published models of pGVHD that also demonstrate lymphocytic pulmonary infiltrates, lung injury, and increased airway resistance [2], [6], [7], [18].

To explore potential mechanisms of lung injury in AlloLPS mice, we compared in detail the lymphocyte subtypes between AlloLPS and SynLPS lungs. We show that the majority of pulmonary T cells in AlloLPS and SynLPS mice are TEM cells with a very small fraction of TN cells. This is consistent with published studies of the T-cell-transfer colitis model where injection of naïve T cells into syngeneic Rag1^−/−^ mice leads to their proliferation and conversion to TEM cells [19], [20]. However, in contrast to syngeneic controls, allogeneic lymphocyte transfer and LPS lead to a striking increase in pulmonary CD8 T cells, including TEM, TN, and effector CD8 T cells. The IL-15 upregulation seen in AlloLPS mice is likely an important pro-CD8 T cell stimulus. Indeed, IL-15 has been shown to cause formation of memory CD8 T cells, CD8 T cell proliferation, and CD8 IFN-γ production post HCT [21], [22]. CD8 T cells have previously been implicated in systemic GVHD pathophysiology [23], [24]. CD8 T cells have also been identified in the pulmonary inflammatory infiltrates after HCT [7], [18], [25]–[27]. While it has not been demonstrated whether CD8 T cells are necessary for post-HCT pulmonary inflammation, allogeneic CD8 T cell activation and proliferation has been shown to occur directly in the airways in response to epithelial alloantigen expression [28]. Our study is unique in showing that the preferential CD8 proliferation and activation occurs in the setting of alloantigen exposure and environmental stimulus, without a full HCT procedure and a conditioning regimen.

Based on previously published studies, CD8 T cells may be the primary mediators of airway injury and AHR pathogenesis in our experiments. Notably, CD8 T cells have been shown to mediate AHR in several animal models of allergic airways disease [29]–[33]. CD8 T cells are also known to produce IFN-γ and granzyme B, which are known to mediate airway injury. Additionally, the CD8-derived cytokine CCL5 has been identified as a potential modulator of AHR [34] [35] [36].

An additional factor that likely contributes to the overall T cell expansion and lung injury in our model is the unopposed CD4 helper T cell effector action with a relative regulatory T cell deficiency. Tregs, in fact, have been shown to decrease AHR in asthma studies [37]. For example, Tregs are capable of moderating systemic and pulmonary inflammation in GVHD [38], [39] as well as in acute lung injury models [40]. Future experiments should focus on whether Treg administration can reverse the lung injury phenotype in our model. The CD4 effector T cell expansion in our experiments is associated with increased IFN-γ, which is consistent with Th1 polarization. In contrast, Th2 cytokines (IL-4, IL-5, and IL-13) are not elevated, suggesting that AHR in this model is not Th2-mediated, contrary to AHR in asthma models. Additionally, the AHR does not appear to be mediated by Th17 cells given the reduced IL-17 levels in AlloLPS lungs [41], [42].

We would like to acknowledge several limitations of our present study. First, exposures to an innate immune stimulus potentiate a prominent localized pulmonary lymphocytic inflammation in both the allogeneic and syngeneic setting. While the abnormal lung pathology in SynLPS mice may have some features of syngeneic GVHD, our model differs from the published models of syngeneic GVHD models that utilize HCT conditioning [43]–[45]. The lymphocytic accumulation in SynLPS lungs appears benign, with little airway injury or T cell activation; we speculate that it is due to homeostatic proliferation previously described in the setting of lymphopenia in the T-cell-transfer model of chronic colitis [19]. Homeostatic proliferation is likely a major driver of lymphocyte expansion in the lungs of both AlloLPS and SynLPS mice and, as such, SynLPS mice serve as a control for this “background” homeostatic proliferation process. However, we acknowledge that our experiments were not designed to specifically assess the role of homeostatic proliferation in AlloLPS lung injury or the mechanisms of the SynLPS pulmonary process. Second, we cannot fully assess the relative contribution of the allogeneic cell transfer versus LPS to the AHR in AlloLPS mice. Given that the allogeneic transfer alone without LPS, and the LPS exposures without cell transfer, lead to minimal pathology, we propose that the two insults are synergistic. Third, the Rag1^−/−^ mice received a transfer of spleen cells, not isolated lymphocytes. This makes it impossible to completely rule out the possibility that allogeneic myeloid cells play a role in the development of airway injury. Nevertheless, there is minimal engraftment of donor-derived myeloid cells at the time point studied, making it unlikely that these cells are important in development of disease. Finally, because IL-15 is also a potent stimulus for NK cell recruitment and NK cells can represent an important source of IFN- γ and granzyme B [46], we measured total NK cells in the lungs of AlloLPS and found them similar to those in SynLPS. However, we cannot rule out the presence and contribution specifically of IFN-γ-producing NK cells or other innate lymphoid cells in AlloLPS disease.

In summary, we demonstrate that allogeneic lymphocyte transfer followed by sub-acute exposure to LPS leads to lung injury and is associated with an increase in CD8 T cells, effector CD4 T cells, and inflammatory cytokines. Furthermore, our results establish a novel model of pGVHD that combines allogeneic and environmental stimuli in the absence of a full HCT and conditioning regimen. Future experiments should focus on specific roles of CD8 T cells and Tregs in the pathogenesis of lung injury in this model of murine pGVHD.

## Supporting Information

Checklist S1 (PDF)
